# The interest of eye‐tracking for apathy detection in patients with Mild Neurocognitive Disorders

**DOI:** 10.1002/alz70856_099728

**Published:** 2025-12-24

**Authors:** Valeria Manera, Maria Cordero‐Rull, Razeen Hussain, Fabio Solari

**Affiliations:** ^1^ CoBTeK Laboratory (Cognition Behaviour Technology), Nice, Alpes Maritimes, France; ^2^ Université Cote d'Azur, CoBTeK lab, Nice, PACA, France; ^3^ DIBRIS, University of Genoa, Genoa, Liguria, Italy

## Abstract

**Background:**

Apathy, defined as a significant reduction in self‐initiated, goal‐directed behaviors, is one of the most prevalent neuropsychiatric symptoms in Neurocognitive Disorders (NCD). Early and accurate detection is critical for implementing timely interventions. Clinicians and researchers emphasize the importance of using objective, quantifiable behavioral biomarkers to complement traditional clinical assessments. Eye‐tracking techniques offer the potential to quantify exploratory behaviors, which could enhance the assessment of apathy. However, few studies have explored the use of eye‐tracking for apathy evaluation.

**Method:**

The study included 20 participants with Mild NCD (mean age = 71.0 years) and 18 participants with subjective cognitive decline (mean age = 68.4 years). Participants were administered two tasks: 1) viewing 9‐second social vs. non‐social videos (featuring people vs. objects), and 2) using the Chosis application, which asks participants to select a picture of their favorite item (e.g., food, leisure activity) from a list. A Tobii eye‐tracker recorded participants' eye movements during the tasks. Apathy was assessed using the Diagnostic Criteria for Apathy and the self‐reported Apathy Motivation Index. A comprehensive neuropsychological assessment was also conducted to evaluate participants' cognitive decline, focusing on memory, attention, working memory, and executive functions.

**Results:**

Preliminary findings indicate that apathetic participants exhibited more fixations and saccades compared to non‐apathetic participants, with shorter fixation durations on average. These results suggest that apathetic individuals may engage in more exploratory behavior when viewing videos and images. Contrary to a hypothesis of diminished behavioral exploration, apathetic participants appear to demonstrate a less organized exploration pattern when viewing videos and scenes. Ongoing correlation analyses with neuropsychological test results are investigating potential links with executive functions.

**Conclusion:**

The exploration of images and videos through eye‐tracking may offer complementary insights into apathy, providing additional information beyond observable behavior. This approach could improve our understanding of apathy mechanisms and enhance its assessment in clinical settings.

